# A Putative Lipid-Associating Motif in the West Nile Virus NS4A Protein Is Required for Efficient Virus Replication

**DOI:** 10.3389/fcell.2021.655606

**Published:** 2021-05-12

**Authors:** Andrea Mikulasova, Leah K. Gillespie, Rebecca L. Ambrose, Turgut E. Aktepe, Alice M. Trenerry, Susann Liebscher, Jason M. Mackenzie

**Affiliations:** ^1^Department of Physiology Anatomy and Microbiology, La Trobe University, Melbourne, VIC, Australia; ^2^Department of Microbiology and Immunology, University of Melbourne, Melbourne, VIC, Australia

**Keywords:** West Nile virus, RNA replication, membrane remodeling, NS4A protein, virus-host interactions

## Abstract

Flavivirus replication is intimately associated with re-organized cellular membranes. These virus-induced changes in membrane architecture form three distinct membranous “organelles” that have specific functions during the flavivirus life cycle. One of these structures is the replication complex in which the flaviviral RNA is replicated to produce progeny genomes. We have previously observed that this process is strictly dependent on cellular cholesterol. In this study we have identified a putative cholesterol recognition/interaction amino acid consensus (CRAC) motif within the West Nile virus strain Kunjin virus (WNV_KUN_) NS4A protein. Site-directed mutagenesis of this motif within a WNV_KUN_ infectious clone severely attenuated virus replication and the capacity of the mutant viruses to form the replication complex. Replication of the mutant viruses also displayed reduced co-localization with cellular markers recruited to replication sites during wild-type virus replication. In addition, we observed that the mutant viruses were significantly impaired in their ability to remodel cytoplasmic membranes. However, after extensive analysis we are unable to conclusively reveal a role for the CRAC motif in direct cholesterol binding to NS4A, suggesting additional complex lipid-protein and protein-protein interactions. We believe this study highlights the crucial role for this region within NS4A protein in recruitment of cellular and viral proteins to specialized subdomains on membrane platforms to promote efficient virus replication.

## Introduction

Replication of flaviviruses, like all positive-stranded RNA viruses, is associated with specialized cytoplasmic membrane structures that wrap around the active replication complexes (RC), providing a favorable microenvironment that facilitates efficient RNA synthesis. The membrane structures induced in flavivirus-infected cells appear especially intriguing in that they consist of at least two or three well defined compartments with distinct functions during replication (Westaway et al., 1997b; Mackenzie et al., 1999; Mackenzie, 2005). Membranes derived from the endoplasmic reticulum (ER) undergo proliferation and remodeling in the process of their formation. In particular they can be observed as a complex network of convoluted membranes (CM) or paracrystalline arrays (PC), or vesicular/spherular invaginations packed within membrane sacs, termed vesicle packets (VP) (Mackenzie et al.,1996a,b; Westaway et al., 1997b; Mackenzie, 2005). Immunogold-labeling, subcellular fractionation and electron tomography studies have revealed that the VP house the flavivirus RC, whereas the role the CM/PC has been postulated to function during translation and proteolytic maturation of the flavivirus polyprotein (Mackenzie et al., 1996a, 1998, 2007a; Westaway et al., 1997b, 1999).

Our detailed ultrastructural analysis, using an Australian WNV strain Kunjin virus (WNV_KUN_) as a model system, allowed us to derive the consensus viral protein composition of the WNV_KUN_ RC. Based on the analysis of purified active membrane fractions and immunoelectron microscopy (cryo-IEM), we identified five of the seven non-structural (NS) proteins (NS1, NS2A, NS3, NS4A, and NS5) involved in replication of the viral RNA (Westaway et al., 1997b, 2002; Mackenzie et al., 1998). NS5 protein contains motifs for methyl transferase and RNA-dependent RNA polymerase (RdRp) motifs (Rice et al., 1985; Koonin, 1993). The remaining NS proteins most likely function as regulatory/accessory molecules and play a role in RC formation, a prerequisite step for productive viral RNA replication. In addition to the role of viral proteins in the formation and integrity of the WNV_KUN_ RC we have also identified an essential requirement for intracellular cholesterol (Mackenzie et al., 2007b). We observed that drug-induced perturbations that reduced the synthesis of intracellular cholesterol drastically impaired WNV_KUN_ replication. This appears to be solely due to the intracellular concentration (or distribution) of cholesterol as treatment of infected cells with methyl-β-cyclodextran did not significantly affect replication *per se*. Subsequently it has been observed that dengue virus replication alters lipid homeostasis (Perera et al., 2012), but is equally dependent on cholesterol, sphingolipid and continual fatty acid synthesis (Rothwell et al., 2009; Heaton et al., 2010; Martin-Acebes et al., 2014). These complementary studies have emphasized the strict requirement of cellular lipids in providing a membrane platform for efficient flavivirus replication.

Since the RC of all positive-sense RNA viruses are dependent on membrane interactions, lipids are expected to play a major role in facilitating viral replication and proliferation. Indeed, these RNA viruses actively modulate cellular lipid metabolism to provide a lipid-rich environment suitable for structural and functional integrity of their RC. Cellular lipid requirements vary between different virus families and individual members, as do the intracellular sites and cellular membranes/organelles utilized during virus replication (Miller and Krijnse-Locker, 2008). In the last years, the role of cholesterol in infection has gained much attention. Studies based on transcriptome and proteomic analyses have demonstrated that the expression of genes related to the synthesis and transport of cholesterol and fatty acids is dysregulated in Hepatitis C virus (HCV) infected cells (Fujino et al., 2010). Moreover, results investigating HCV replication using statins (inhibitors of cholesterol synthesis) and other compounds specific for inhibition of fatty acid biosynthesis, have clearly shown that a decrease in cellular fatty acid and cholesterol biosynthesis reduces HCV replication (Su et al., 2002; Ye et al., 2003). Recent investigations of flavivirus-infected cells have revealed that dengue virus (DENV)-infected cells have altered lipid homeostasis (Perera et al., 2012), increased fatty acid synthesis (Heaton et al., 2010) and appear to degrade lipid droplets to release free fatty acids (Heaton and Randall, 2010). In addition, it was recently shown that WNV also alters lipid biosynthesis and requires sphingolipid metabolism for efficient production of WNV virions (Martin-Acebes et al., 2014). These recent studies provide increasingly evidence for the role of lipids in the replication of the *Flaviviridae*.

In this study we have extended our previous finings to define a potential role for cholesterol-rich micro-domains within the ER in facilitating WNV_KUN_ RC formation and function. We have also identified a region within the N-terminus of the WNV_KUN_ protein NS4A that contributes to this. To this end we have performed site-directed mutagenesis within a WNV_KUN_ infectious clone to inactivate a potential membrane proximal cholesterol recognition/interaction amino acid consensus (CRAC) motif identified within NS4A. We investigated the importance of the CRAC motif on virus replication by generating recombinant viruses containing alternations in this motif.

## Materials and Methods

### Viruses and Cells

Cells were infected with WNV_KUN_ strain MRM61C at an approximate multiplicity of infection (m.o.i.) of 3 as has been described previously (Westaway et al., 1997a). Vero and BHK cells were maintained in DMEM supplemented with 5% FCS (Lonza, Basel, Switzerland) and penicillin/streptomycin (100 U/mL and 100 μg/mL, respectively, GIBCO-BRL) at 37°C with 5% CO_2_.

### Antibodies

WNV_KUN_ specific anti-NS1 (clone 4G4; Macdonald et al., 2005) and anti-NS5 (clone 5H1.1) monoclonal antibodies were generously provided by Dr. Roy Hall (University of Queensland, Brisbane, Australia). WNV_KUN_-specific rabbit anti-NS4A polyclonal antisera has been described previously (Mackenzie et al., 1998). Rabbit anti-β-1,4-galactosyltransferase (GalT) polyclonal antibodies (Berger et al., 1993) were generously provided by Dr. Eric Berger (University of Zurich, Zurich, Switzerland). Rabbit anti-Erlin-2 antibodies were purchased from Cell Signaling Technologies and mouse anti-dsRNA (clone J2) antibodies were purchased from English & Scientific Consulting Bt. (Hungry). Alexa Fluor 488- and 594-conjugated anti-rabbit and anti-mouse specific IgG were purchased from Molecular Probes (Invitrogen, Leiden, Netherlands).

### Plasmids and Transfection

Recombinant cDNA plasmids expressing eGFP-tagged erlin-1, erin-2, prohibitin, stomatin, and flotillin were kindly provided by Dr. Stephen Robbins (University of Calgary, Canada). Plasmids were introduced into cells via Lipofectamine delivery following the manufacturer’s instructions.

### Immunofluorescence (IF) Analysis

Vero cell monolayers on coverslips were infected with WNV_KUN_ and incubated at 37°C for 24 h. The cells were subsequently washed with PBS and fixed with 4% paraformaldehyde (Sigma Aldrich, St. Louis, MO) and permeabilized with 0.1% Triton X-100 as previously described (Malet et al., 2007). Primary and secondary antibodies were incubated within blocking buffer (PBS containing 1% BSA) and washed with PBS containing 0.1% BSA between incubation steps. After a final wash with PBS the coverslips were drained and mounted onto glass slides with a quick dry mounting medium (United Biosciences, Brisbane, Australia) before visualization on a Leica TCS SP2 confocal microscope. Images were collected using a Leica digital camera and Leica 3D software before processing for publication using Adobe Photoshop^TM^ software. Colocalization was quantified based on the fluorescence microscopy images and was performed using ImageJ software via the colocalization analysis plug-in JACoP. Images were collected from replicate experiments with at least 20 cells counted from each.

### Site-Directed Mutagenesis of WNV_KUN_ (FLSDX) Infectious Clones

Tyrosine to serine (Y/S), at position 28 within NS4A, or lysine to leucine (K/L), at position 35, or double mutations (Y/S + K/L) were generated in the WNV_KUN_ cDNA infectious clone, FLSDX (Khromykh et al., 1998), using site-directed mutagenesis (Stratagene). All clones were sequenced prior to maxiprep amplification (Invitrogen) and transfection.

### *In vitro* Transcription and Electroporation

All replicon templates were linearized with *Xho*I (New England BioLabs) and purified using Phenol/Chloroform extraction and ethanol precipitation. *In vitro* RNAs were transcribed using 1 μg linearized DNA template using established methods previously described (Khromykh and Westaway, 1997; Khromykh et al., 1998), except introduction of the *in vitro* transcribed RNAs into mammalian cells was performed via Lipofectamine-mediated delivery or electroporation via the Neon^TM^ transfection system (Invitrogen) following the manufacturers instructions. Briefly, Vero or BHK cells (1.0 × 10^7^) were electroportated using a 100 μL tip, at 1,300 volts, width 20, and 2 pulses. Cells were resuspended in cell culture media, seeded, and incubated for various periods for examination of virus replication.

### Plaque Assay

Vero C1008 cells were seeded in DMEM complete media in 6-well plates and incubated at 37°C overnight. Virus stock was diluted 10-fold in 0.2% BSA/DMEM and cells were infected with 300 μL of stock dilutions (in duplicate) and incubated at 37°C for 60 min. Two milliliter of a semi-solid overlay containing 0.3% w/v low-melting point agarose, 2.5% w/v FCS, 1% Penicillin/Streptomycin, 1% Glutamax, 1% HEPES and 0.1% NaCO_3_ was added to cells and solidified at 4°C for 30 min. Cells were incubated at 37°C for 3 days, fixed in 4% v/v formaldehyde (in PBS) for 1 hour and stained in 0.4% crystal violet (with 20% v/v methanol and PBS) at RT for 1 hour. Plaques were manually counted and plaque-forming units per mL (pfu/mL) calculated.

### Western Blotting

Transfected cells were aspirated in PBS then lysed in SDS lysis buffer (0.5% SDS, 1 mM EDTA, 50 mM Tris-HCl) containing protease inhibitors leupeptin (1 μg/mL) and PMSF (0.5 mM) and phosphatase inhibitors sodium orthovanadate (25 mM), sodium fluoride (25 mM) and β-glycerophosphate (25 mM) (Sigma). Lysates were diluted in LDS loading buffer (Invitrogen), heated at 70°C for 5 min and separated on a 10% Tris-Glycine polyacrylamide gel. Proteins were transferred to Hi-Bond ECL nitrocellulose membrane (Amersham Biosciences) and the membrane was blocked with 5% w/v skim milk (Diploma) in TBS with 0.05% Tween (PBS-T). Primary antibodies were incubated at 4°C with membrane overnight in blocking solution as above. Following primary incubation, the membrane was washed in TBS-T then incubated with secondary antibodies conjugated to Cy5 (Amersham Biosciences), Alexa Fluor 647 or Alexa Flour 488 (Invitrogen) in TBS-T at RT for 2 h. The membrane was washed twice in TBS-T then TBS, and proteins visualized on the Storm Fluorescent scanner (Amersham Biosciences) on either 635 nM or 430 nM emission channel.

### Resin Thin Sections for Electron Microscopy

Cells were fixed with 3% glutaraldehyde in 0.1 M cacodylate buffer for 2 h at room temperature. Cells were washed several times in 0.1 M cacodylate buffer followed by fixation with 1% OsO4 in 0.1 M cacodylate buffer for 1 h. After washing of the cells in 0.1 M cacodylate buffer, specimens were dehydrated in graded acetones for 10–20 min each. Subsequently, samples were infiltrated with EPON resin and polymerized in molds for 2 days at 60°C. 50–60 nm thin sections were cut on a Leica UC7 ultramicrotome using a Diatome diamond knife and collected on formvar-coated copper mesh grids. Before viewing in a JEOL 2010 transmission electron microscope cells were post-stained with 2% aqueous uranyl acetate (UA) and Reynold’s lead citrate.

### Transient Expression of Mutant NS4A Proteins, Immune-Precipitation and Assessment of Cholesterol Content

Tyrosine to serine (Y/S), at position 28 within NS4A, or lysine to leucine (K/L), at position 35, or double mutations (Y/S + K/L) were transferred from the mutant FLSDX to a pcDNA3.1 NS4A(-2K)-6xHis expression construct. Briefly, the NS4A-2K amplicon was amplified from FLSDX using forward (ATGGGCCCACCATGTCTCAAATAGGT) and reverse (TAT TTCTAGACTAATGGTGATGGTGATGGTGGCGTTGCTTCT CTGGCTCAGG) primers and ligated into pcDNA3.1 using *Eco*RV and *Xba*I (Promega). Following propagation and midiprep purification (QIAGEN), 5 μg of DNA was transfected into 293T cells using Lipofectamine 2000 (Life Technologies) as indicated by manufacturers. At 24 h.p.t, cells were lysed on ice in a cholesterol extraction buffer (50 mM Tris pH 8.0, 150 mM NaCl, 0.1% w/v Digitonin, 0.1% NP-40, 0.5% Triton-X 100 and 0.25% sodium deoxycholate) containing a protease inhibitor cocktail (Astral Scientific), and centrifuged at 10,000 rcf to pellet cellular debris. Immunoprecipitation was performed on the lysates using an anti-His6 antibody (Abcam) coupled with Protein A Sepharose (Life Technologies) to pull-down the expressed NS4A-2K proteins. Lysates and IP samples were then assayed for cholesterol content using the Amplex Red cholesterol assay kit as indicated by the manufacturers. Total cholesterol (in μg) was calculated using internal standard curves and error bars indicate ± 1 standard deviation (*n* = 4).

### Statistical Analyses

Data is representative of 3 independent experiments and was analyzed by unpaired Student’s *t-*test using GraphPad Prism v8.0.

## Results

### WNV_KUN_ RC Is Localized Within Cholesterol-Rich Micro-Domains Within the Membranes of the ER

**FIGURE 1:**
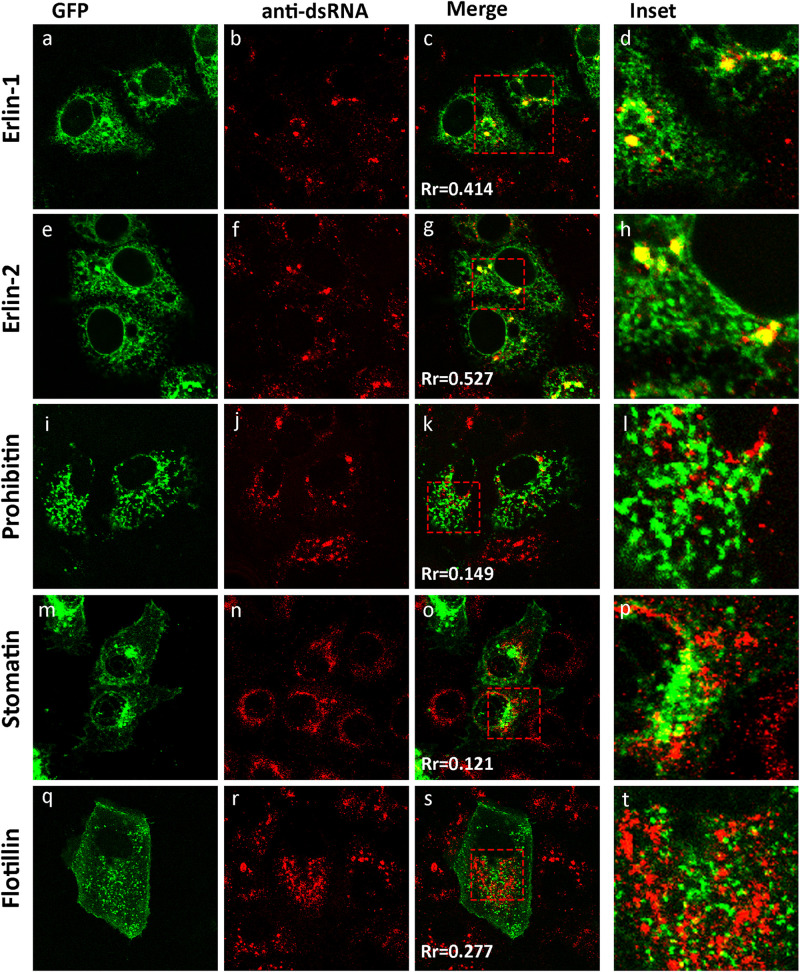
The WNV_KUN_ RC resides within cholesterol-rich domains in the ER. IF analysis of Vero cells transfected with recombinant eGFP-expression plasmids for 24 h **(a,e,i,m,q)** and subsequently infected with WNV_KUN_ for an additional 24 h. Cells were immuno-stained with antibodies to dsRNA, and co-stained with Alexa Fluor 594 **(b,f,j,n,r)**. Prominent co-localization between dsRNA and erlin proteins is observed as a yellow hue in **(c,h)** only. Rr is the Pearson’ co-efficient **(c,g,k,o,s)** as assessed in Image J and serves as a quantitative measure of co-localization. Values above 0.4 indicate a significant degree of co-localization **(d,h,l,p,t)**. Represents the inset for the red box in **(c,g,k,o,s)**, respectively.

Previously we have shown that WNV_KUN_ redistributes intracellular cholesterol to the sites of viral RNA replication, the VP (Mackenzie et al., 2007b). In addition, we have observed that the biogenesis of the VP appears to occur on a membrane platform derived from the ER, and recruitment of both host and viral proteins occurs at a pre-Golgi step (Gillespie et al., 2010). To further characterize the interactions that occur during development of the VP we utilized two GFP-tagged protein markers, erlin-1 and erlin-2, originally used to define cholesterol-rich micro-domains within the ER (Browman et al., 2006). Both proteins fall within the growing family of prohibitin domain-containing (PHB) proteins and are selectively targeted into ER micro-domains in a cholesterol dependent manner (Browman et al., 2006). We examined subcellular distribution of ectopically expressed eGFP-tagged erlin-1/2 proteins within the ER membranes in WNV_KUN_-infected Vero cells. Immunofluorescence (IF) analysis indicated that a significant pool of erlin-1/2 was confined to the cytoplasmic foci recognized by anti-dsRNA antibody (Figures 1a–h). The specific accumulation of erlin proteins within WNV_KUN_ replication sites was further confirmed by observation that other PHB family members, with different intracellular targets, did not co-localize with anti-dsRNA antibody stained foci (Figures 1i–t). In agreement with published findings, eGFP-tagged versions of prohibitin-1, flotillin-1 and stomatin-1a, were found to inhabit lipid micro-domains in the mitochondria (Figure 1i) and in endosomes and the plasma membrane (Figures 1m,q), respectively, quite distinct from WNV_KUN_ dsRNA.

These results suggest that WNV_KUN_ replication is associated with cholesterol enriched membrane domains within the ER, and co-located with the host proteins erlin-1 and 2.

### Attenuated Replication Rates of WNV_KUN_ Recombinant Viruses Carrying Mutation in NS4A CRAC Motif

**FIGURE 2:**
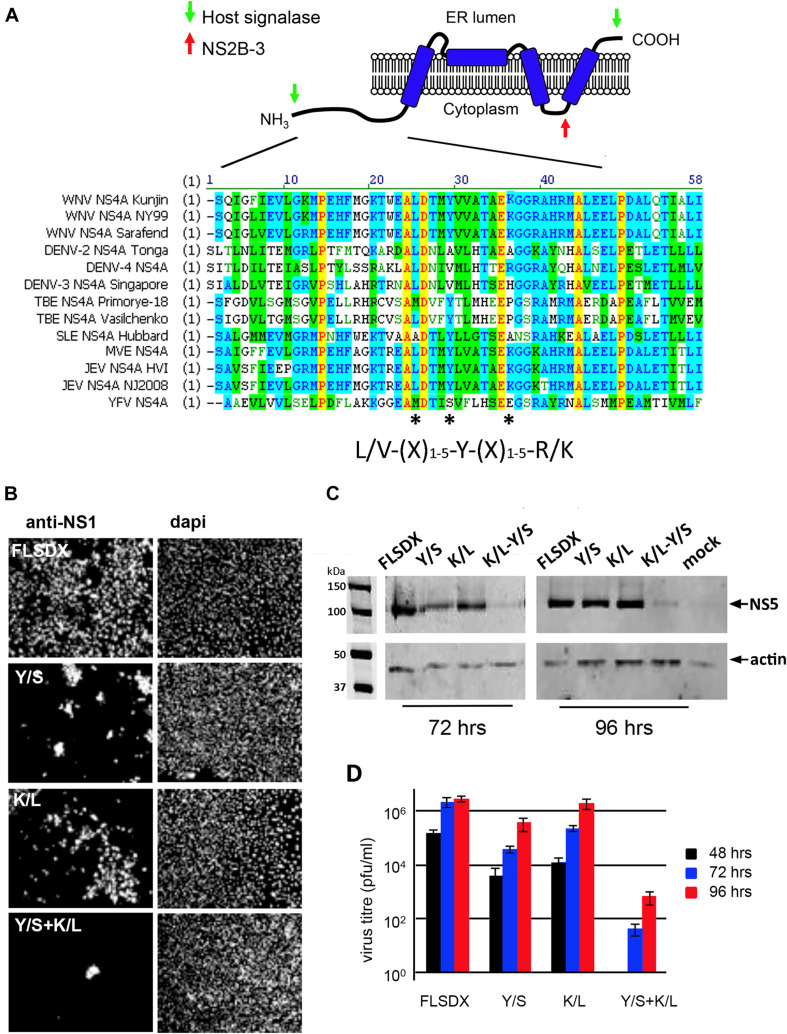
**(A)** WNV_KUN_ NS4A protein encodes for a potential CRAC motif. NS4A ^25^L-(X)-^29^Y-(X)-^36^K region is highly conserved within the Japanese encephalitis subgroup of the *flavivirus* genus. Various NS4A sequences from the *flavivirus* genus were aligned using ClustalW software (Vector NTI; Invitrogen). Light gray residues are conservative and the amino acids comprising the CRAC motif are highlighted with asterisks (*). The predicted topology of NS4A in the endoplasmic reticulum membrane is modified based on the study by Miller et al. (2007). **(B)** Mutation of the NS4A CRAC motif restricts WNV_KUN_ replication. Single (Y/S or K/L) and double residue (Y/S + K/L) mutations were generated in the CRAC motif in FLSDX using site-directed mutagenesis. RNA was transcribed and transfected into Vero cells and replication was assessed by IF analysis at 72 h.p.e. with antibodies raised against the WNV NS1 protein and counterstained with the nuclear dye dapi. **(C)** In addition, protein lysates were collected at 72 and 96 h.p.e. and analyzed by Western blotting using the antibodies raised against WNV NS5 protein and compared to the internal control actin. **(D)** Tissue culture fluid was collected at 48, 72, and 96 hpe and each time point was analyzed by plaque assay, and visible plaques were used to calculate titers for FLSDX and the Y/S, K/L and Y/S + K/L mutants. Error bars indicate standard deviation derived from replicate experiments (*n* = 3).

As we had observed previously and above; cholesterol appears to play a significant role in the biogenesis and establishment of the WNV_KUN_ RC (Figure 1; Mackenzie et al., 2007b) we aimed to identify whether a specific viral gene product is actively involved in lipid recognition/and redistribution. Our initial studies focused on the WNV_KUN_ NS4A protein as we, and others, had previously observed that flavivirus NS4A has the capacity to remodel intracellular membranes (Roosendaal et al., 2006; Miller et al., 2007). To this end, we utilized gene mining and revealed that NS4A contains a potential cholesterol recognition/interaction amino acid consensus (CRAC) motif [L/V^24^-X_(__1–5__)_-Y^28^-X_(__1–5__)_-R/K^35^] motif near its’ N terminus (Figure 2A). Sequence alignments revealed that this motif is highly conserved within members of the Japanese Encephalitis subgroup, with limited homology within the other members of the *flavivirus* genus (Figure 2A). A CRAC motif has been identified in the peripheral-type benzodiazepine receptor (Li and Papadopoulos, 1998) and other proteins known to bind cholesterol, including caveolin-1 (Parton et al., 2006), human immunodeficiency virus (HIV) gp41 (Vishwanathan et al., 2008) and the influenza M2 protein (Schroeder et al., 2005). Although it should be noted that presence of a CRAC motif does not solely confer the ability of a protein to associate with cholesterol (e.g., caveolin and cholesterol binding; Epand et al., 2005). To interrogate the importance of the CRAC domain on virus replication we generated recombinant viruses containing inhibitory mutations within this motif in the NS4A protein. Mutations were made within the WNV_KUN_ infectious clone (FLSDX) to alter residues within NS4A at position 28 from tyrosine to serine (Y/S), or to change residue at position 35 from lysine to leucine (K/L). Mutation of these amino acids was previously observed to disrupt cholesterol binding and trafficking of the major myelin protein P0 (Saher et al., 2009) and influenza virus M2 protein (Thaa et al., 2011).

Recombinant viruses harboring corresponding single or combined double mutations (Y/S + K/L) exhibited attenuated phenotypes varying in degrees (Figure 2B), of which the double mutation was shown to be extremely attenuated and was difficult to analyze even after subsequent re-infection. Viral replication efficiency was assessed by IF, Western blotting and plaque assay in Vero cells transfected with RNAs transcribed *in vitro* from FLSDX cDNAs (Figures 2B,D). It was observed that recombinant viruses harboring the K/L mutation displayed a slight delay in growth kinetics and was moderately attenuated, as assessed by plaque assay (Figure 2D). Whereas recombinant viruses harboring the Y/S mutation were more attenuated than the K/L and WT viruses. We failed to recover any of the double mutant in Vero cells. However, we could detect basal virus protein production and infectious virus release in BHK cells that were electroporated with the viral Y/S + K/L RNA (data not shown). Sequencing analysis of the recovered viruses revealed that no compensatory mutations had occurred, even after prolonged incubation of the transfected cells (data not shown).

These results indicated that the potential CRAC motif within the WNV_KUN_ NS4A protein appeared to play a major role during the replication cycle. The results also indicated that conservation of Tyr at position 28 is critical for efficient replication of the WNV_KUN_ genome and double mutation of Tyr-28 and Lys-35 is lethal to WNV_KUN_ replication in Vero cells.

### WNV_KUN_ Replication Complex Formation Is Significantly Impaired Upon Transfection With CRAC Mutant Viruses

**FIGURE 3:**
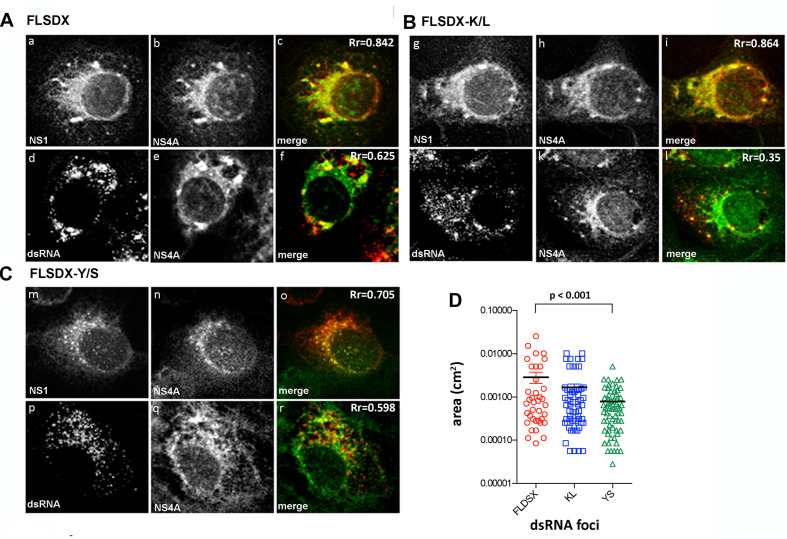
Mutation within the CRAC motif prevents WNV_KUN_ RC formation. Vero cells were transfected with FLSDX WT **(A)**, FLSDX K/L **(B)**, or FLSDX Y/S **(C)** RNAs and at 48 h.p.e. fixed for IF analysis. Transfected cells were then immune-stained with antibodies specific for WNV NS1 (in red) and NS4A (in green) or dsRNA (in red) and NS4A (in green). Images were collected on a confocal microscope; and yellow in the merge images indicates co-localization. **(D)** the size (area) of the dsRNA foci were analyzed by Image J software and statistical significance was determine by Student’s *t-*test.

To determine the stage in the replication cycle affected by the Y/S and K/L mutants, we assessed the ability of these constructs to form the WNV_KUN_ RC by IF analysis. Vero cells were electroporated with the individual CRAC mutants and at 48 h.p.e., localization and distribution of the RC was determined by immuno-staining for dsRNA and NS4A or NS1 and NS4A (Figure 3). For both wild-type (FLSDX) and the K/L mutant, dsRNA and NS1 were detected in individual and often sizable foci scattered throughout the perinuclear/reticular area of the cytoplasm (Figures 3Aa–c,g–i), that largely coincided with NS4A (Figures 3Ad–f,Bj–l). Comparatively in the case of the Y/S mutant, dsRNA and NS1 were further dispersed in smaller foci (Figures 3Cm–o,D) and there was a reduction in observable co-localization between the dsRNA and NS4A (Figures 3Cp–r). These results suggested that RC formation was significantly impaired in the Y/S mutant viruses.

**FIGURE 4:**
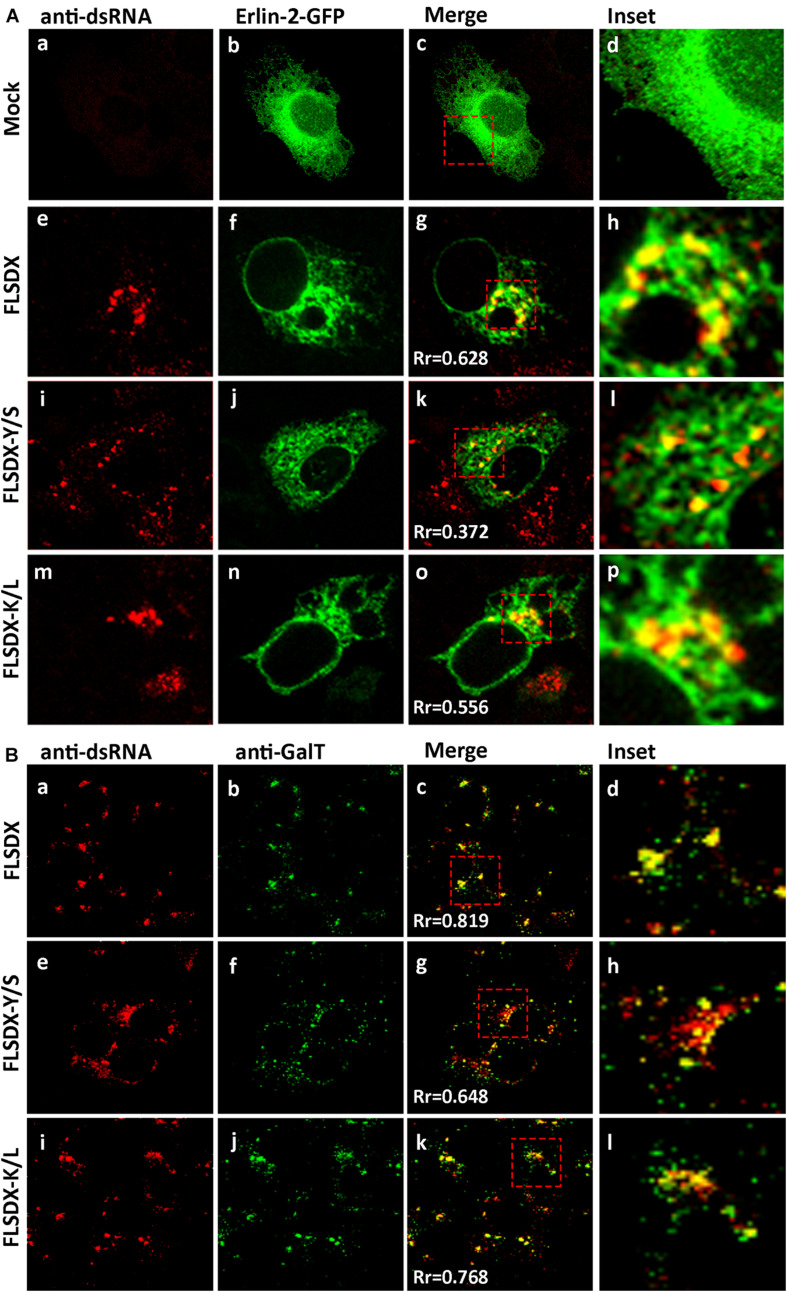
CRAC mutant FLSDX viruses are impaired in their ability to recruit host proteins to the WNV_KUN_ RC. **(A)** Vero cells transfected with recombinant eGFP-erlin-2 plasmids for 24 h and subsequently electroporated with CRAC mutant FLSDX viruses for an additional 48 h. Cells were immuno-stained with antibodies to dsRNA, and co-stained with Alexa Fluor 594 (panels **a, e, i**, and **m**). Prominent co-localization between dsRNA and eGFP-erlin-2 proteins (panels **b,f,j**, and **n**) is observed as a yellow hue in the merge and insert panels **c–d**, **g–h**, **k–l** and **o–p**. **(B)** Cells were immune-stained with antibodies specific to dsRNA and counterstained with species specific Alexa Fluor 594 (panels **a, e**, and **i**), and with antibodies specific to GalT and counterstained with Alexa Fluor 488 (panels **b,f,** and **j**). Co-incidental labeling is depicted as a yellow hue in the merged and insert panels on the right hand side (panels **c–d**, **g–h**, and **k–l**).

As previously shown, WNV_KUN_ replication is associated with cholesterol enriched membrane domains in the ER (Figure 1), and recently YFV replication was observed to localize to detergent resistant membranes high in cholesterol content (Yi et al., 2012). Thus, we aimed to further characterize the defect in RC formation by assessing whether the Y/S and K/L mutants had retained the ability to localize to cholesterol-rich domains within the ER, as marked by the host protein erlin-2. Vero cells were electroporated with mutant genome RNAs and the eGFP-tagged erlin-2 plasmid DNA to define cholesterol-rich domains in the ER (Figure 4A). At 48 h.p.e., localization and distribution of the RC was determined by immuno-staining for dsRNA. In transfected and mock-infected cells, eGFP-erlin-2 was observed to localize in a typical reticular ER-like pattern without any noticeable cytoplasmic foci (Figures 4Aa–d). For both FLSDX and the K/L mutant, dsRNA was confined to large reticular/perinuclear cytoplasmic foci where eGFP-erlin-2 was significantly co-located (Figures 4Ae–h,m–p). However, we observed that eGFP-erlin-2 and dsRNA were dispersed in visibly smaller foci throughout entire cytoplasm of the cell and revealed reduced co-localization in cells replicating the Y/S mutant (Figures 4Ai–l). These results suggested that there was an impairment of the WNV_KUN_ Y/S mutant to localize to lipid microdomains in the ER to establish biogenesis of the RC, an impairment that correlates with attenuation of replication efficiency (Figure 2).

To further investigate the defect in RC formation we assessed whether it coincided with a defect in recruitment of cellular proteins known to localize to the RC (Mackenzie et al., 1999). Vero cells were electroporated with mutant genome RNAs and at 48 h.p.e., localization of the RC and *trans*-Golgi network glycoproteins was determined by immunostaining for dsRNA and GalT (Figure 4B). For both FLSDX and the K/L mutant, dsRNA and GalT colocalized in foci in the perinuclear/reticular region of the cytoplasm (Figures 4Ba–d,i–l). Comparatively in the case of the Y/S mutant, dsRNA and GalT were dispersed in significantly smaller foci throughout the cytoplasm and there was a reduction in the co-localization between the two (Figures 4Be–h). These results highlighted that there was dissociation between cellular and viral components that are normally found within the RC in the Y/S mutant viruses.

Overall, our results suggest that the CRAC motif within the WNV_KUN_ NS4A protein appears to play a major role facilitating efficient virus replication. In particular that the Y/S mutation significantly impaired the capacity of the mutant viruses to effectively form the WNV_KUN_ RC complex, which may be related to the inability of mutant viruses to unite the required viral and cellular replicative components to specific subdomains on the ER membrane.

### WNV_KUN_-Induced Membrane Proliferation Is Significantly Impaired Upon Transfection With CRAC Mutant Viruses

**FIGURE 5:**
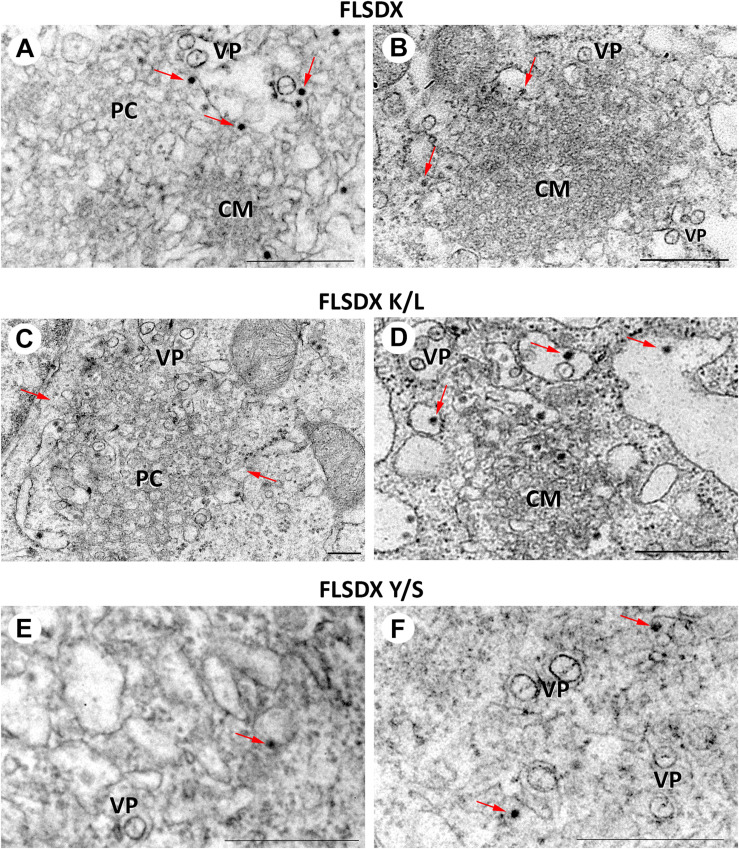
Mutation of the CRAC domain within the WNV_KUN_ NS4A proteins results in reduced membrane remodeling. Ultrastructural analysis of Vero cells electroporated with CRAC mutant FLSDX *in vitro* transcribed RNAs (FLSDX, **A,B**; K/L mutant, **C,D**; Y/S mutant, **E,F**) at 48 h.p.i. Characteristic flavivirus-induced membrane structures convoluted membranes (CM) and paracrystalline arrays (PC) are only observed in FLSDX- and K/L mutant-electroporated cells. However, vesicle packets (VP) and virus particles (arrows) are observed in all cells. Magnification bars represent 200 nm.

During WNV_KUN_ infection ER membranes undergo extensive proliferation and rearrangements such that they can be observed as an intricate network of CM/PC and VP via ultrastructural analysis (Mackenzie et al., 1996a; Westaway et al., 1997b; Mackenzie, 2005). The CM/PC is thought to function during translation and proteolytic maturation of the polyprotein, whereas the VP is known to house the RC (Mackenzie et al., 1996a, 1998, 2007a; Westaway et al., 1997b, 1999). In view of the defect in RC formation in the Y/S mutant observable via IF analysis, we were interested to investigate whether the similar characteristic membrane structures, particularly the VP, were induced in these mutant viruses. Thus, Vero cells were electroporated with the individual CRAC mutants, fixed at 48 h.p.e. and analyzed by electron microscopy (Figure 5). Interestingly, VP formation was decreased but observable in the Y/S mutants in comparison to the FLSDX and K/L mutants. More strikingly, however, was that recombinant viruses harboring the Y/S mutation were observed to be significantly impaired for CM/PC induction; the induced CM/PC structures were abundant and could clearly be defined in the FLSDX and K/L mutants but were almost absent in the Y/S mutants (compare panels in Figure 5). All mutant viruses appeared to produce virus particles, albeit greatly reduced in the Y/S mutant. No evidence of infection was observed for the double mutant.

These results indicated that the CRAC motif within the WNV_KUN_ NS4A protein appeared to play a major role in facilitating membrane proliferation and remodeling.

### The CRAC Motif Promotes Colocation of NS4A With Erlin-2 and a Potential Association With Cellular Cholesterol

**FIGURE 6:**
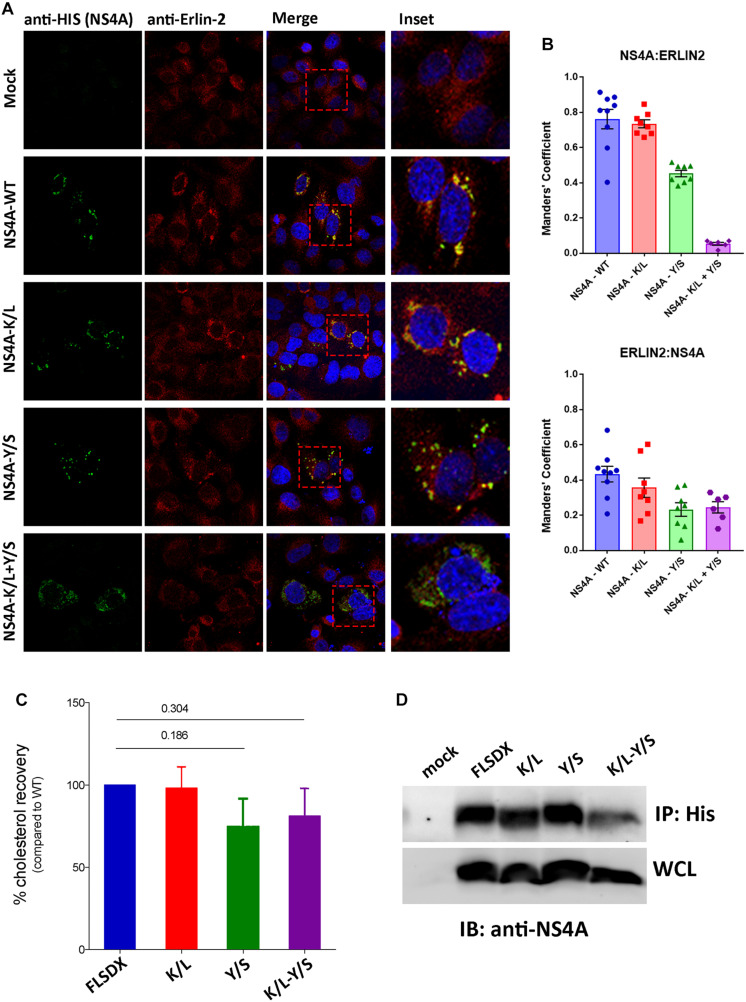
The CRAC motif promotes colocation of NS4A with erlin-2 and a potential association with cellular cholesterol. **(A)** Vero cells transfected with recombinant NS4A-His plasmids for 24 h and subsequently immuno-stained with antibodies to 6xHis co-stained with Alexa Fluor 488 with antibodies to erlin-2 and co-stained with Alexa Fluor 594. Prominent co-localization between dsRNA and eGFP-erlin-2 proteins is observed as a yellow hue in the merge and insert panels on the right-hand side. **(B)** The extent of co-localization was assessed in Image J by evaluating the Manders’ coefficient of NS4A-erlin-2 and erlin-2-NS4A. **(C)** Percentage amount of cholesterol recovered from transfected cells after immune-precipitation with anti-His antibodies. Cholesterol recovery was measured via the Amplex Red cholesterol assay kit. Statistical significance was determined by Student’s *t-*test. **(D)** A representative western blot showing the expression level of the NS4A mutants in the input whole cell lysate (WCL) and their recovery after IP. Western blot was probed with anti-NS4A antibodies.

During our studies we could not directly observe co-location of NS4A with endogenous erlin-2 due to conflict with the same species of antibodies. As an alternative to understand this we transfected Vero cells with cDNA expression plasmids expressing the individual NS4A fused to a 6xHIS tag and co-stained those cells with anti-erlin-2 antibodies (Figure 6A). As can be observed expression of WT and all single NS4A mutants induced the formation of cytoplasmic foci that co-located strongly with erlin-2 for WT and NS4A K/L and to a lesser degree for NS4A Y/S (quantitation provided in Figure 6B as Manders’ co-efficient). In contrast, the NS4A K/L + Y/S mutant displayed a more diffuse cytoplasmic staining with little co-location with erlin-2 (Figure 6A).

To address whether the CRAC motif within NS4A conferred a biochemical interaction with cellular cholesterol we expressed the WT and mutant NS4A proteins transiently in 293T cells (due to increased transfection efficiency). Our initial analysis to co-localize intracellular cholesterol (using filipin or perfringolysin-O) with NS4A was unsuccessful (data not shown). Therefore, we aimed to isolate NS4A and determine if we could co-purify cholesterol (or not) after immune-precipitation. As observed in Figure 6D we could immune-purify all forms of the NS4A protein and could interact with a similar percent of cholesterol, as determined by the Amplex Red cholesterol assay kit, with the WT and K/L mutant NS4A (Figure 6C). In contrast we observed less cholesterol isolated with the Y/S and K/L + Y/S mutant NS4A proteins, however, these results were not significant even after repeated experiments (Figure 6C; *n* = 4). At this stage we cannot conclusively determine whether the potential CRAC motif is promoting an association with cholesterol or whether other domains within NS4A or additional protein-protein or protein-lipid interactions are occurring.

Overall, we can conclude that the CRAC motif is important for WNV_KUN_ replication and appears crucial for the ability of NS4A to co-locate with erlin-2 within microdomains within the ER to remodel intracellular membranes and promote WNV_KUN_ RC assembly and function. Whether cholesterol is indeed a contributing factor is still unresolved.

## Discussion

WNV_KUN_ replication, like all flaviviruses is intimately linked with membrane induction and remodeling. The WNV_KUN_ RC itself is formed on a membrane platform derived from the ER and is the site where RNA is replicated to produce progeny viral genomes (Gillespie et al., 2010). Our previous studies have revealed that this process is reliant on cellular cholesterol (Mackenzie et al., 2007b). In this study we extended these findings to define a potential role for cholesterol-rich micro-domains within the ER in facilitating the biogenesis and function of the WNV_KUN_ RC. Previously, our mutational analyses have revealed that conserved amino acids within the N-terminus confer stability of the NS4A important in membrane remodeling enabling virus replication (Ambrose and Mackenzie, 2015). Here we have extended those studies to show that a putative CRAC domain also within the N-terminus of the WNV_KUN_ NS4A protein appears to facilitate efficient virus replication within these modified membranes. Mutation of this domain within NS4A significantly impaired coalescence of both viral and cellular factors on the ER membrane to promote efficient virus replication.

Within this report we have extended our list of host proteins co-localizing within the WNV_KUN_ RC and show that two host proteins known to associate with “lipid raft-like,” cholesterol-rich domains within the ER, erlin-1 and erlin-2, co-locate with dsRNA during infection (Figure 1). The association of erlin-1 and erlin-2 with the RC strengthens a role for cholesterol in the biogenesis and maintenance of the WNV_KUN_ RC. Additionally it should be noted that a recent proteomic analysis revealed an association of erlin-2 with DENV NS5 protein (Carpp et al., 2014). Previously we had shown that the WNV_KUN_ RC could be stained with filipin and chemical modulation of cholesterol homeostasis duly affected virus RNA replication (Mackenzie et al., 2007b). In addition, it was recently observed that the host protein DNAJC14, a protein known to reside within detergent-resistant (lipid-raft-like) membranes, was recruited to the Yellow fever virus RC (Yi et al., 2012). Therefore, we suggest that early after translation the flavivirus proteins accumulate within cholesterol-rich patches within the ER, defined by erlin proteins and/or DNAJC14, to promote the biogenesis of the RC. Upon changes in cholesterol concentration, the mobility of proteins within and to these domains, maybe altered impacting on RC formation and thus replication efficiency. It is also pertinent to note that our previous studies revealed a very close association of the CM/PC structures with the cholesterol-synthesizing enzyme 3-hydroxy-methyglutaryl-CoA reductase, HMGCR (Mackenzie et al., 2007b). These individual observations strongly implicate a role for cholesterol and cholesterol synthesis during the flavivirus replication cycle.

We extended these observations further to identify factors responsible for co-ordinating localization within these cholesterol-rich domains within the ER and gene mining identified a potential lipid-binding (CRAC) motif within the N-terminus of NS4A, which is highly conserved within members of the Japanese Encephalitis subgroup of *flaviviruses* (Figure 2A). To interrogate the role of this motif during replication we undertook a mutagenesis analysis of this CRAC motif within NS4A. Our investigations revealed that recombinant viruses harboring a double mutation (Y/S + K/L) were significantly impaired in their ability to replicate effectively, so much so that replication was extremely difficult to analyze even following subsequent re-infection (Figures 2B–D). The individual K/L mutant was slightly attenuated at earlier time points as demonstrated by IF analysis, viral titer and protein levels, whilst the Y/S mutant was severely attenuated (Figures 2B–D). Our analyses also revealed that the Y/S mutant was particularly impaired in its ability to form the RC, unite viral and cellular proteins that house within the RC, target to cholesterol-rich domains within the ER and the capacity to remodel cytoplasmic membranes (Figures 3–6). We observed that the Y/S virus replication sites, identified by anti-dsRNA antibodies, were smaller in size compared with the parental FLSDX virus and much more diffuse within the cytoplasm. Additionally, we observed reduced co-localization between dsRNA and NS4A and between the replication proteins NS1 and NS4A in cells transfected with the Y/S mutants (Figure 3). This was comparatively reflected in the ability of the Y/S mutant to recruit the host proteins GalT and erlin-2 within the RC (Figure 4). In cells transfected with the Y/S mutant GalT maintained a more perinuclear staining pattern consistent with localization within the Golgi apparatus, whereas erlin-2 was more diffusely localized within the cytoplasm. However, it should be noted that we were unable to conclusively demonstrate that the CRAC motif facilitates a direct interaction between NS4A and cholesterol (Figure 6), and thus requires further studies to delineate how the RC is formed on these lipid sub-domains. For example, formation of the RC maybe driven via an interaction between NS4A and DNAJC14 or between NS4A and erlin proteins on the ER membrane. It should also be noted that we recently reported that this region of the NS4A protein mediates an interaction with the membrane-bending host protein Reticulon 3A.1 (Aktepe et al., 2017). We also cannot rule out or discount other cellular responses, such as the unfolded protein response, that could also influence membrane proliferation and alteration. These are all areas we are currently investigating.

In addition, to biogenesis of the RC our EM analysis revealed that the CRAC motif within NS4A contributed significantly to the capacity of NS4A to remodel cytoplasmic membranes characteristic of flavivirus infection (Figure 4). We observed that cells transfected with the Y/S mutant still produced virus particles and VP, albeit at greatly reduced numbers compared to the K/L and FLSDX viruses. The most notable observation was the complete absence of CM/PC membranous structures in the Y/S transfected cells. We, and others, have shown that NS4A has the capacity to remodel cytoplasmic membranes and induce the formation of CM/PC when expressed alone (Roosendaal et al., 2006; Miller et al., 2007). The results presented here would suggest that the putative CRAC motif is potentially the region within NS4A responsible for this remodeling, a proposal we are currently investigating. The question still remains how the absence of the CM/PC contributes to impaired RNA replication and facilitates the recruitment of host proteins to the VP? We have previously prosed that the CM/PC are the intracellular site of efficient viral protein translation and processing, and our current observations may indicate that they are equally involved with sorting proteins and membrane within the infected cell. It is possible that during established replication viral proteins (and RNA) destined for the VP must in fact transit the CM/PC before delivery, thereby ensuring that only the correct proteins (and lipid) reside within the RC. In the situation observed with the Y/S mutant this obviously does not occur and as such viral proteins remain associated with unmodified ER and thus cannot unite to generate the CM/PC and VP. Although we did observe some VP in our EM analyses of the Y/S mutant, we suggest that this may simply be a situation where the required elements (viral and cellular proteins and lipid) are in the correct vicinity during translation and thus can invoke some limited membrane remodeling.

Overall, we have shown that a conserved motif within the N-terminus of the WNV_KUN_ NS4A protein enables it to remodel cytoplasmic membranes and recruit both viral and host cell proteins to cholesterol-rich microdomains within the ER that facilitate the process of RC biogenesis. Whether this motif is directly responsible for cholesterol recruitment though still remains to be elucidated. Newly developed approaches such as photo-activable cholesterol and mass-spectrometry could enhance to sensitivity and specificity of detection. In the end, this study highlights that a functionally competent NS4A is critical to the formation and remodeling of membrane structures associated with the WNV RC and to recruit both viral and host cell proteins to these structures. The study supports and extends previous reports from ourselves and other, of the importance of the N-terminus of the flavivirus NS4A in facilitating virus replication.

## Data Availability Statement

The raw data supporting the conclusions of this article will be made available by the authors, without undue reservation.

## Author Contributions

AM, LG, RA, TA, and AT performed the experiments. AM, LG, RA, TA, SL, AT, and JM collated and assembled the data. RA, SL, TA, and JM wrote the manuscript. All authors contributed to the article and approved the submitted version.

## Conflict of Interest

The authors declare that the research was conducted in the absence of any commercial or financial relationships that could be construed as a potential conflict of interest.
